# Escape MD: Using an Escape Room as a Gamified Educational and Skill-Building Teaching Tool for Internal Medicine Residents

**DOI:** 10.7759/cureus.18314

**Published:** 2021-09-27

**Authors:** Aakanksha Khanna, Adharsh Ravindran, Brandon Ewing, Karen Zinnerstrom, Connor Grabowski, Archana Mishra, Regina Makdissi

**Affiliations:** 1 Department of Internal Medicine, Jacobs School of Medicine and Biomedical Sciences, Buffalo, USA; 2 Office of Medical Education, Jacobs School of Medicine and Biomedical Sciences, Buffalo, USA

**Keywords:** higher education medical training, medical education med ed learning classroom integrated, student education, teaching strategies, medical education & training, general radiology

## Abstract

Purpose

To create an innovative medicine-themed escape room (EsR) and assess its feasibility as a learner-centered educational model for medical trainees. This platform could be used to teach and reinforce medical knowledge as well as enhance team-building skills.

Materials and Methods

We created an internal medicine (IM) themed EsR, in which participants are locked and instructed to solve a series of puzzles using both medical and nonmedical concepts to "escape" the room within a given set of time. The players must use their critical thinking and communication skills to solve puzzles consisting of complex activities (such as image identification and object matching or retrieval) linked in a nonlinear pattern. A pre-activity survey was used to collect basic demographic information and initial perceptions of the activity. A post-activity survey consisting of a modified Likert scale and free-response questions was used to assess perceived activity use and satisfaction. The activity was followed by a debriefing session with a faculty member to reflect on individual and team-based learning. This study was approved by the Institutional Review Board.

Results

Each week, a group of four to seven residents participated in a one-hour long EsR session, which was replicated 15 times over five weeks, for a total of 86 internal medicine residents. 76 of 86 residents completed the post-activity survey. Overall, residents expressed a high level of satisfaction with the session (x̄ = 4.89), found it fun to play (x̄ = 4.89), and felt immersed in medicine (x̄ = 3.95). Residents thought the activity was most suitable for reinforcing knowledge (x̄ = 4.26) and greatly tested their communication skills (x̄ = 4.48).

Conclusion

The medical EsR experience was enjoyed by the vast majority of residents with very positive oral and survey feedback. Hence, we successfully created an active, learner-centered, gamified teaching tool that can be used for teaching/reinforcing medical concepts in a fun, competitive, and team-building format. The EsR, as a teaching tool, can be replicated with ease several times and requires very few resources to create.

## Introduction

A shift is needed from a teacher-centered approach to a learner-centered approach in medical education. New research in interactive teaching models has forced educators to try activities such as a flipped classroom or Jeopardy to enhance learning. "Gamification," defined as the use of game design elements in a non-game context, has become a popular instructional method in K-12 and higher education [1]. However, there is limited research using the efficacy of gamification in medical education.

Escape rooms (EsRs) have gained traction in popular culture as an activity that fosters teamwork and peer group communication. Conventionally, EsRs are defined as a competitive mental and physical adventure game, carried out in a confined room and constrained by time, where a team of participants must discover clues and solve a mystery to escape a locked room. When used in the clinical context, EsRs are considered a form of gamification [2]. In a non-medical context, the current focus of gamification revolves around either enhancing general interpersonal skills such as teamwork or communication or targeting specific skill sets necessary to carry out a particular intervention such as a particular procedure [3]. Playing a game creates a dynamic educational environment that enhances retention of knowledge, encourages bonding, improves communication, and reinforces learning [4-5]. Well-designed games can often encourage "stealth learning," where learners often are unaware of the skills they have acquired until assessment after the activity [3]. Unlike traditional teaching methods, gamification enables learning to be optimized towards an individual's or peer groups’ strengths [3]. Proper targeting of peer-group learning ultimately enables the development of leadership, empathy, and teamwork skills through real-life practice and application [4]. Furthermore, this platform provides an opportunity to teach team-building skills in a psychologically safe environment. Of note, there is no consensus on the best method for addressing the Accreditation Council for Graduate Medical Education (ACGME) core competencies such as communication and teamwork skills, competencies that directly affect resident training and education [6]. Some institutions have sought to develop active-learning techniques, such as mock code blue, in an attempt to teach their trainees these skills [7]. Additionally, the reception of these activities tends to remain highly dependent upon a variety of factors, including the quality of the facilitators and standardization [7-8].

It is important to note that there have been some attempts to bring such activity to the education realm for pharmacy, dermatology, and radiology students [9-15]. However, these attempts were limited in sample size, most were conducted outside the United States, and they generally did not involve medical students or physicians-in-training. Research is lacking in using the EsR room as a platform for medical trainees for education and team-building purposes. Thus, there is a need for research into the effect of play on an individual's ability to flourish in the medical field.

Our goal was to create a novel internal medicine-themed activity and assess its feasibility as a team-based, competitive medical teaching tool that can be used as an adjunct to traditional teaching practices in a psychologically safe and fun environment. Our future goal is to expand this platform in a variety of in-person and virtual ways to create learner- and/or topic-specific sessions that can be incorporated into the traditional teaching curriculum.

## Materials and methods

Study design

The EsR activity was created as a mandatory teaching session for all of our trainees. Approval for the survey study was obtained from the Institutional Review Board at the State University of New York at Buffalo. A pre-activity survey was used to collect demographic data on participants, information regarding general learning style, and initial perceptions of the activity. Pre-activity survey data was anonymous. A post-activity survey based on a modified five-point Likert scale, along with several free-response questions was used to analyze participant perceptions after the activity. An attention check question was placed at the end of the survey to ensure residents were truly reading each question.

Resources

No external funding was provided for this study. We did not obtain any grants for this project. The games were created by A. Khanna and A. Ravindran. The budget was set by the program director and approved using the residency department funds.

Participant selection

Our internal medicine program works on a 4+1 schedule (four weeks of rotations followed by one week of ambulatory clinic week). Approximately 20 residents were placed into one of five clinic cohorts. A group of four to seven residents from each cohort was randomly assigned to one of three group activities. The activity was conducted once a week, for five consecutive weeks, in place of a traditional didactic lecture. All residents (postgraduate years 1-3) needed to attend the activity, but residents on vacation during their ambulatory week were excused. 

Game design and framework

**Figure 1:**
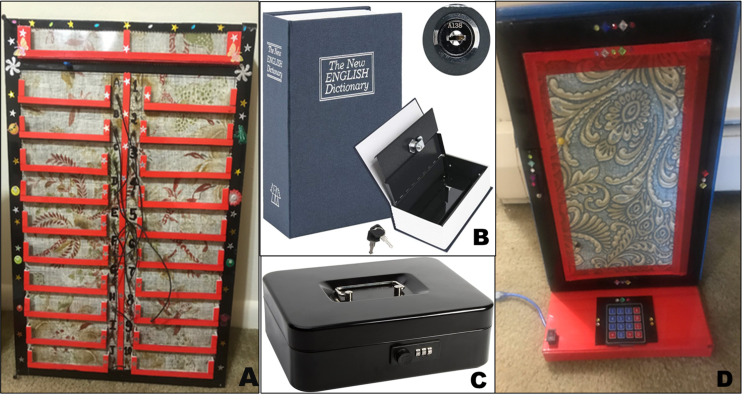
Sample games used in the escape room A) Device used to facilitate a medicine-themed matching game. The slots on the left host a series of riddles printed on laminated cards, while the opposing column contains the answers. Each slot is associated with an inlet for an auxiliary wire. Upon correctly wiring the slots containing the riddles on the left with the answers on the right, the device displays a clue, allowing the game to continue. B) Lockbox disguised as a dictionary. Upon completion of the activity in panel A, participants are able to unlock this box containing a series of laminated electrocardiogram rhythms, one of which has an additional clue hidden on its back, directing participants to the next activity. C) An additional lockbox found in the room. Completion of the activity described for panel B enables participants to unlock this lockbox. D) Lockbox device used in the final circuit (IV) of the game. The front of the device contains a slot holding a sheet of laminated paper with a specific scenario accompanied by a multistep question. The base of the device contains a digital keypad. Each answer forms a component of the three-digit code needed to open the device.

Broadly two categories of games, mental and physical, were designed to use critical thinking skills, logic, dexterity, and communication skills to create a fun learning experience. Games included a series of increasingly complex puzzles that were interlinked in a linear pattern to form one circuit. Upon completion of each puzzle, an item or clue was given, which would take the participants to the next puzzle within that circuit. The general framework for the EsR consisted of three circuits of puzzles, each with a specific objective in mind, that would come together at the end to form the final circuit. Circuits one and two were independent of one another and could be initiated at any given time. Circuit three could only be started if certain components circuit one and circuit two were discovered. At the end of each circuit, participants received an item; the participants would collect a total of three items that would allow them to unlock the final circuit. Two distractor puzzles were also incorporated within the circuits. Examples of the types of devices used in the EsR are shown in Figure 1.

**Figure 2:**
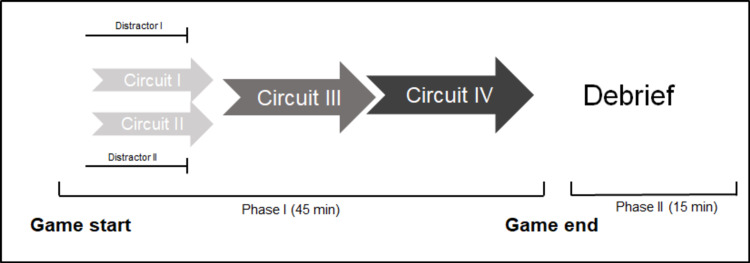
Framework of the escape room

The general framework for the EsR consisted of three circuits of puzzles, each with a specific objective in mind, that would come together at the end to form the final circuit. Circuits one and two were independent of one another and could be initiated at any given time. Circuit three could only be started if certain components of circuit one and circuit two were discovered. At the end of each circuit, participants received an item; the participants would collect a total of three items that would allow them to unlock the final circuit. Two distractor puzzles were also incorporated within the circuits. A schematic depicting the general framework of the escape room is seen in Figure 2.

Room design

**Figure 3:**
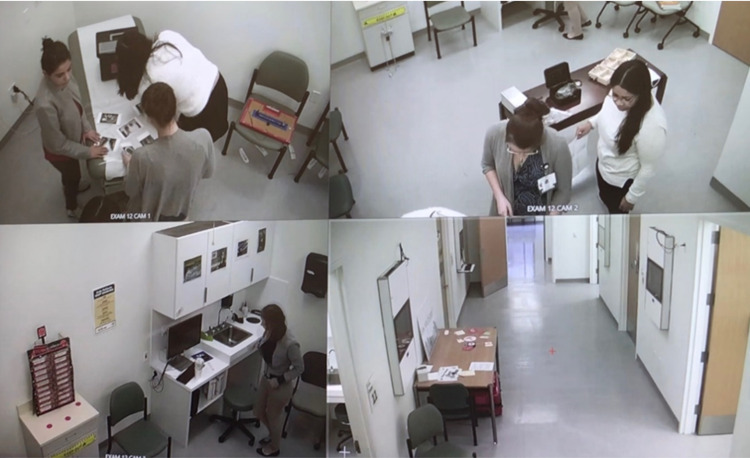
Faculty observing game play of our participants via a control room

Before the start of the activity, participants were given the chance to complete a 2-min (optional) pre-activity demographic survey as well as signed a consent form regarding participation. Game Master, a member of the team, introduced participants to the rules and gave them a medicine-themed story for the EsR. Participants were given 45 min to complete the activity. During the activity, participants were watched through multiple cameras, and observations were recorded (Figure 3).

Participants were provided hints along the way if they were stuck on a puzzle for more than 10 mins. Upon completion of the activity, a 15-min debriefing was conducted by our program’s director or associate program director to reflect on individual and team-based learning. This opportunity was utilized to identify knowledge gaps, reflect on strengths and discuss ways to improve teamwork. A post-activity survey was available on Google© forms (Google, Mountain View, California). 

Topic selection

**Figure 4:**
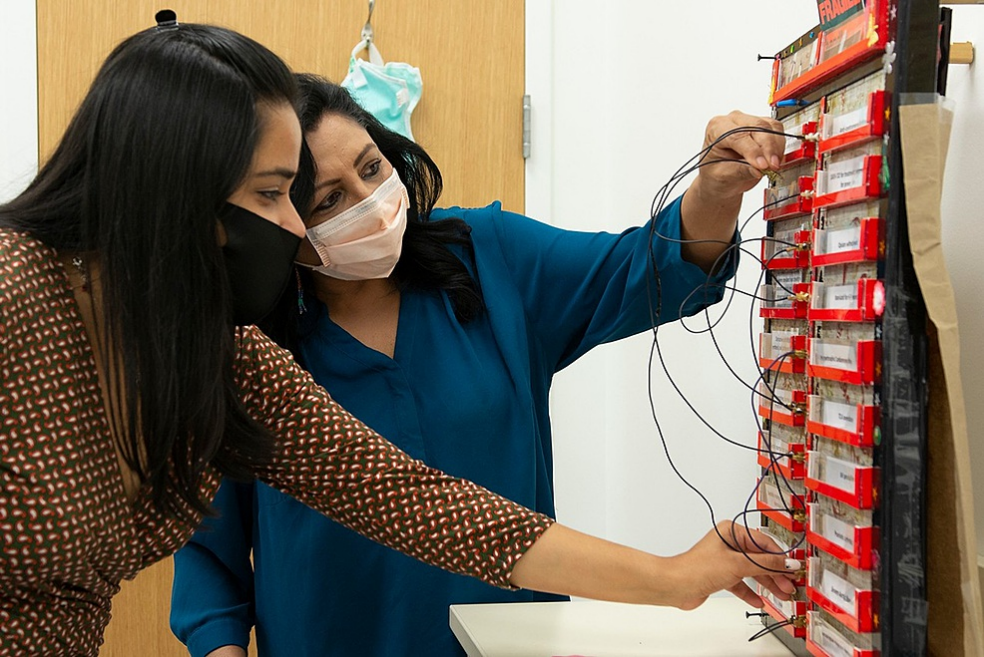
Image depicting team-building behaviors

Topic selection was under the direction of A. Khanna, A. Ravindran, and R. Makdissi. A variety of general medicine topics important for succeeding in an inpatient and outpatient setting were used to create several games and puzzles. These topics included but were not limited to diagnostic skills based on history and physical electrocardiogram interpretation (such as differentiating atrial flutter from atrial fibrillation and multifocal atrial tachycardia), United States Preventive Services Task Force guidelines for screening (such as low-dose computed tomography for a smoker), etc. These topics were included in puzzles as well as American Board of Internal Medicine style case-based questions, and subsequently interlinked together to form the EsR framework as described above. An image depicting team-building behaviors is seen in Figure 4. 

## Results

Demographics

A total of 86 internal medicine residents participated in the mandatory EsR activity as part of their training. 79 residents answered the pre-activity survey. 46/79 (58.2%) identified as males. 32/79 (40.5%) were in their first year of training, 21/79 (26.6%) were in their second year, and 26/79 (32.9%) were in their third year.

Approximately half of the participants (40/79, 50.6%) had attended an EsR or a similar experience in the past, in a non-medical setting. 

Prior to the the activity, most of the participants indicated that they were visual-type learners (27/79, [34.2%]), where as 29.1% indicated that they were kinesthetic learners (learn best by doing), followed by verbal (16/79, 20.3%), social (7/79, 8.9%), and auditory learnings (6/79, [7.6%]). When asked about the most common resources used to study, the majority indicated it was a combination of UpToDate (UpToDate Inc, Waltham, Massachusetts) (60/79, [75.9%]), question banks (34/79, [43.0%]), and textbooks (34/79, [43%]). 

Of interest, the majority of the participants (56/79, 70.9%) indicated that they were interested in exploring new ways of learning. 

Escape room feedback

76/86 participants completed the post-survey. Three residents did not answer the attention question correctly at the end of the post-survey, and their responses were excluded. Residents expressed a high level of satisfaction with the activity as a whole (x̄ = 4.89) and found the overall session fun to play (x̄ = 4.89). They indicated the information used to solve the puzzles was similar to information needed in general medicine services (x̄ = 3.78). Additionally, they thought the activity was most suitable for reinforcing knowledge (x̄ = 4.26) and were able to test their ability to communicate with one another (x̄ = 4.48). Table 1 further illustrates the results of the post-activity survey data.

**Table 1:** Post-survey results x̄ is defined as the average value on a 1-to-5 scale, with 1 being defined as the lowest possible value and 5 being defined as the highest possible value

General information	x̅
Overall, I enjoyed the activity	4.89
I found the game fun to play	4.89
Preparation and event experience	
I read other resources to prepare for this activity	3.93
The challenges in the game were interesting to me	4.57
I found the event to be stressful	1.75
The information I needed to solve puzzles was similar to the information I need to use in the internal medicine department	3.78
I learned from my peers during the escape room	4.32
The questions asked were appropriate for the setting	4.5
The format would be an appropriate method for reinforcing knowledge	4.26
Learning	
The large-group debriefing and discussion following this activity helped me to better understand the impact of individual contributions on teamwork and communication	4.46
This activity was successful in testing my individual ability to communicate effectively within a team	4.48
This format helped me identify knowledge gaps	3.75
Application of event	
The format would be an appropriate medium for teaching new information	3.75
I found the non-educational portions of the activity helped me learn the content	3.69
The format would be an appropriate method for testing knowledge	3.42
If I were to participate in another event similar to this one, I would feel motivated to prepare for it	3.65
There should be more games of this type integrated into medical training	4.38
I would recommend this activity to other students	4.74

**Figure 5:**
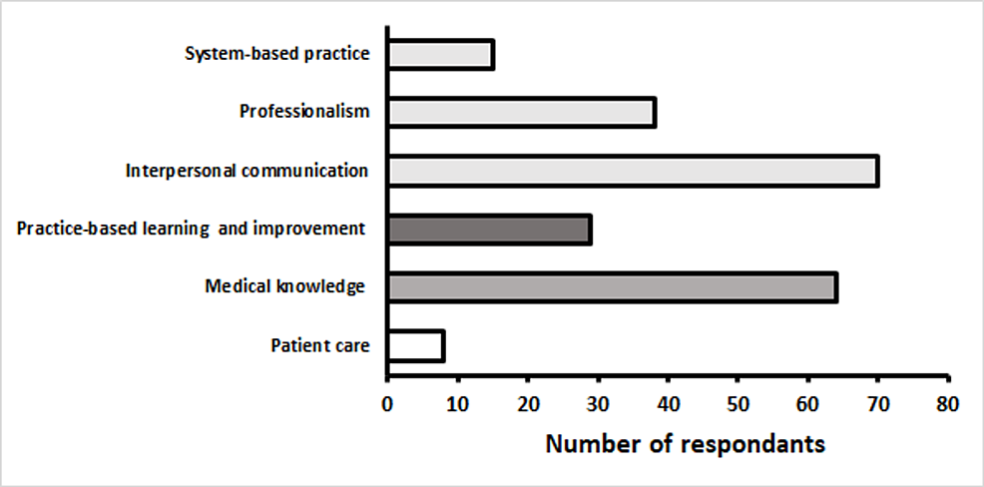
Participants' answers (on a choose-all-that-apply style of question) on the ACGME core competencies used ACGME - Accreditation Council for Graduate Medical Education

When asked to choose all the ACGME core competencies that were used during the EsR, residents most frequently reported using interpersonal communication and medical knowledge (Figure 5).

## Discussion

We were able to create and replicate several educational EsR activities for our residents and students in a low-cost, high-impact, nontraditional teaching session. Overall, the experience was enjoyed by the vast majority of our participants. As summarized by Liu et al., reception to similarly designed rooms is generally well-received [9]. However, contrary to other studies, our target population comprised those at the postgraduate level. During the debriefing session, residents frequently mentioned that they enjoyed the opportunity to connect with one another and thought that the residency curriculum, in general, did not give them this opportunity. The free-response data on our post-activity survey confirmed this trend, where the most cited ACGME core competency associated with the room (on a choose-all-that-apply style question) was team bonding and communication, a forgotten but important aspect to success during residency. Additionally, the questions for our particular room generally involved knowledge that preclinical students may not necessarily be acquainted with at their level. 

Despite the vast number of learning styles indicated on the pre-implementation survey, the residents generally agreed that the activity promoted retention of information and identification of knowledge gaps. It is important to state that residents did not have a strong opinion on whether the activity would be appropriate for teaching or testing new information. It is plausible that this could change depending upon the types of questions asked during the activity; however, the majority of residents indicated in the post-activity survey that questions were indeed suitable for the activity. The results also suggest that ample opportunities were given to not only teach but also assess the critical aspects of all six ACGME general competencies. As intended, residents discovered meaningful connections between the activity and everyday clinical life situations, such as leading a medical emergency team.

The success of our EsR has led us to explore additional avenues for implementation. Because the original activity was designed to be flexible and scalable to any difficulty level, we postulated that the activity would also be a useful adjunct for fourth-year medical students. As described by Slavin et al., the final year of medical school, especially in the spring, offers great flexibility for students but is often underutilized. Hence, it is reasonable to believe an EsR activity could also introduce students to skills necessary for residency without overburdening them during their intern year [16]. Although the assessment of the EsR is ongoing, the narrative feedback has been overwhelmingly positive. We are also exploring the creation of EsRs specific for fellows, as many of these skills can apply across all levels.

It is important to recognize the limitations of our study. First, our study was primarily descriptive in nature. We did not include a control arm, and so we cannot definitely conclude whether this activity is more enjoyable or effective than other teaching methods listed in our pre-implementation survey, and we cannot verify our question with statistical certainty. As this was a preliminary study, the goal was to see whether this form of activity is enjoyed by our target population. In different populations, other studies have found evidence suggesting statistically significant increases in knowledge retention immediately after their respective activities [9, 12]. Furthermore, Liu et al. reported that knowledge from their EsR persisted at a two-week follow-up [9]. Nevertheless, the primary conclusion that can be made from this study in regards to information retention or learning effectiveness is based on the residents’ perception of their performance. Hence, it can only be said that the residents perceived the activity as a potential means of reinforcing knowledge. To date, no studies have examined how the EsR fairs in comparison to traditional didactic lectures. This study has enabled us to delve into the world of using game-based teaching for medical trainees. Upon request, we can provide a mast guide that allows educators to replicate similar experiences for their target audience.

## Conclusions

The EsR was considered an enjoyable activity by the residents. We were able to design an experience that was not only interesting but also academically appropriate for our residents. Our results ultimately indicate that the EsR could be used as an interactive teaching tool, which would act as an adjunctive method to reinforce knowledge and foster team-building skills during residency. Further research should be conducted to investigate whether the EsR is as or more effective for retaining information than other traditional or active-learning methods.
